# Metagenomic Identification of a Novel Salt Tolerance Gene from the Human Gut Microbiome Which Encodes a Membrane Protein with Homology to a *brp*/*blh*-Family β-Carotene 15,15′-Monooxygenase

**DOI:** 10.1371/journal.pone.0103318

**Published:** 2014-07-24

**Authors:** Eamonn P. Culligan, Roy D. Sleator, Julian R. Marchesi, Colin Hill

**Affiliations:** 1 Alimentary Pharmabiotic Centre, University College Cork, Cork, Ireland; 2 School of Microbiology, University College Cork, Cork, Ireland; 3 Department of Biological Sciences, Cork Institute of Technology, Cork, Ireland; 4 Cardiff School of Biosciences, Cardiff University, Cardiff, United Kingdom; 5 Department of Hepatology and Gastroenterology, Imperial College London, London, United Kingdom; Miami University, United States of America

## Abstract

The human gut microbiome consists of at least 3 million non-redundant genes, 150 times that of the core human genome. Herein, we report the identification and characterisation of a novel stress tolerance gene from the human gut metagenome. The locus, assigned *brpA*, encodes a membrane protein with homology to a *brp*/*blh*-family β-carotene monooxygenase. Cloning and heterologous expression of *brpA* in *Escherichia coli* confers a significant salt tolerance phenotype. Furthermore, when cultured in the presence of exogenous β-carotene, cell pellets adopt a red/orange pigmentation indicating the incorporation of carotenoids in the cell membrane.

## Introduction

Metagenomics provides a culture-independent means to access and study the genetic content of all of the microorganisms in a particular environmental niche. Metagenomic analysis can be sequence-based or functional (or a combination of both). The development of faster, cheaper and more accurate next-generation sequencing (NGS) technologies has allowed new insights into microbial community structure and diversity and has led to the discovery of many novel genetic loci [1]–[4]. Functional metagenomics has also been utilised to identify many novel functions through cloning and heterologous expression of metagenomic DNA and subsequent phenotypic detection of a desired trait conferred on the cloning host. Some notable examples include genes encoding proteins of industrial, pharmaceutical and medical relevance such as lipases, esterases and novel antibiotics [5]–[8].

The human gut microbiome has become perhaps the most intensively studied environment using metagenomics [9], [10]. Collectively, there are at least 150 times as many genes in the human gut microbiome than there are human genes in the genome, a large proportion of which are uncharacterised [11]. The ability to respond and adapt to external environmental stresses is key to microbial survival and it is possible to use metagenomics to identify novel mechanisms that enable such survival [12]. In the gastrointestinal (GI) tract microorganisms are faced with numerous challenges such as low pH, low iron concentrations, increased osmolarity, bile, immunity mechanisms and competing microbes [13], [14]. Different sets of genes are activated in response to environmental cues [15]. Work in our lab is focused on genes that confer increased tolerance to osmotic stress [16]. The response to osmotic stress is broad and encompasses many diverse cellular processes and systems [17]. Metagenomics makes it possible to identify novel systems unrelated to the classical (and comprehensively studied) primary and secondary responses of potassium (K^+^) uptake and osmoprotectant utilisation [18]–[20]. We have previously identified a number of novel salt tolerance loci from the human gut microbiota using a combination of functional metagenomic screening, next-generation sequencing and bioinformatic analyses [21]–[23].

In this study we report the identification of a novel salt tolerance gene from a human gut metagenomic library we have previously screened [22]. An *in silico* analysis revealed the gene (which we have termed *brpA*) encoded a putative carotenoid modifying enzyme with homology to a *brp*/*blh*-family β-carotene 15,15′-monooxygenase protein, which cleaves β-carotene to two molecules of *all*-*trans* retinal (vitamin A aldehyde) [24], [25]. Finally, we demonstrate that *brpA* confers an increased salt tolerance phenotype when heterologously expressed in *Escherichia coli.*

## Materials and Methods

### Bacterial strains and growth conditions

Bacterial strains and plasmids used in this study are listed in Table 1. Oligonucleotide primers (synthesised by Eurofins, MWG Operon, Germany) are presented in Table S1. *E. coli* EPI300::pCC1FOS (Epicentre Biotechnologies, Madison, WI, USA) was cultured in Luria-Bertani (LB) medium containing 12.5 µg/ml chloramphenicol (Cm) and in 12.5 µg/ml chloramphenicol plus 50 µg/ml kanamycin (Kan) following EZ-Tn*5* transposon mutagenesis. *E. coli* MKH13 was grown in LB and LB supplemented with 20 µg/ml Cm for strains transformed with the plasmid pCI372. *E. coli* strains containing the pBAD expression vector were cultured in the presence of 100 µg/ml ampicillin.

**Table 1:** Bacterial strains and plasmids.

Strain, plasmid or transposon	Genotype or characteristic(s)	Source or reference
**Strains**		
*E. coli* EPI300	F^−^ *mcrA* Δ(*mrr-hsd*RMS*-mcrBC*) Φ80d*lacZ*ΔM15 Δ*lac*X74 *recA*1*endA*1 *araD*139 Δ(*ara, leu*)7697 *galU galK* λ^−^ *rpsL nupG trfA dhfr*;high-transformation efficiency of large DNA	Epicentre Biotechnologies, Madison, WI, USA
SMG 6	EPI300 containing pCC1FOS fosmid with ∼34 kb of metagenomicDNA from human gut microbiome	This study
SMG 6-EZTn*5* #24	Transposon insertion in gene 24 (which precedes acyltransferase gene *atfA*)	This study
SMG 6-EZTn*5* #26	Transposon insertion in *brpA* gene	This study
SMG 6-EZTn*5* #34	Transposon insertion in acyltransferase gene (*atfA*)	This study
SMG 6-EZTn*5* #38	Transposon insertion in *brpA* gene	This study
*E.coli* MKH13	MC4100*Δ*(*putPA*)101D(*proP*)2D(*proU*)	[26]
*E. coli* MKH13::pCI372	MKH13 containing empty pCI372 plasmid	This study
*E. coli* MKH13::pCI372-*brpA_S_*	MKH13 containing pCI372 with *brpA_S_* gene from SMG 6; “S” subscriptdenotes shorter predicted gene with TTG start codon	This study
*E. coli* MKH13::pCI372-*brpA_L_*	MKH13 containing pCI372 with *brpA_L_* gene from SMG 6; “L” subscriptdenotes longer predicted gene with ATG Start codon	This study
*E. coli* MKH13::pCI372-*brpAatfA*	MKH13 containing pCI372 with *brpAatfA* genes from SMG 6	This study
*E. coli* EPI300::pBAD-*brpA_S_*	EPI300 containing pBAD with *brpA_S_* gene from SMG 6	This study
*E. coli* MKH13::pCI372-*brpA_L_*	MKH13 containing pCI372 with *brpA_L_* gene from SMG 6; “L” subscriptdenotes longer predicted gene with ATG Start codon	This study
*E. coli* MKH13::pCI372-*brpAatfA*	MKH13 containing pCI372 with *brpAatfA* genes from SMG 6	This study
*E. coli* EPI300::pBAD-*brpA_S_*	EPI300 containing pBAD with *brpA_S_* gene from SMG 6	This study
*E. coli* EPI300::pBAD-*brpA_L_*	EPI300 containing pBAD with *brpA_L_* gene from SMG 6	This study
*E. coli* EPI300::pBAD-*brpAatfA*	EPI300 containing pBAD with *brpA* and *atfA* genes from SMG 6	This study
**Plasmids**		
pCI372	Shuttle vector between *E. coli* and *L. lactis*, Cm^R^	[27]
pCC1FOS	Fosmid cloning vector, Cm^R^	Epicentre Biotechnologies, Madison, WI, USA
pBAD	L-arabinose inducible expression vector, Amp^R^	Invitrogen, USA
**Transposon**		
EZ-Tn*5*<*ori*V/KAN-2>	Hyperactive Tn*5* transposon, Kan^R^, inducible high copyorigin of replication – *ori*V	Epicentre Biotechnologies, Madison, WI, USA

Cm^R^, Kan^R^ and Amp^R^ = chloramphenicol, kanamycin and ampicillin resistance respectively.

For growth in minimal media, strains were grown in M9 (Fluka) minimal salts supplemented with final concentrations of 0.4% glucose, 0.2% casamino acids, 2 mM magnesium sulphate (MgSO_4_) and 0.1 mM calcium chloride (CaCl_2_). When required, stock solutions of β-carotene were added to media at a final concentration of 20 µM. Growth media was supplemented with 1.5% agar for plate assays. All overnight cultures were grown with shaking at 37°C.

### Construction and screening of the human gut metagenomic library

A previously constructed fosmid clone library, created from metagenomic DNA from the human gut microbiome [28] was used to screen for salt-tolerant clones. The library was screened using the protocol outlined by Culligan et al [22]. Briefly, a total of 23,040 library clones were screened on LB agar supplemented with 6.5% (w/v) NaCl using a Genetix QPix 2 XT™ colony picking/gridding robotics platform. Plates were incubated at 37°C for 2–3 days and checked periodically for growth of likely salt-tolerant clones.

### Sequencing and bioinformatic analysis

The fosmid insert from clone SMG 6 was fully sequenced and assembled by GATC Biotech (Konstanz, Germany) using the GS-FLX 454 pyrosequencing (Roche) platform on a Titanium mini-run. The full sequence of SMG 6 can be found in GenBank under the accession number JQ269599.1. Putative open reading frames were predicted using Softberry FGENESB bacterial operon and gene prediction software (www.softberry.com) and also GeneMark [29]. Retrieved nucleotide and translated amino acid sequences were functionally annotated by homology searches using the Basic Local Alignment and Search Tool (BLAST) to identify homologous sequences from the National Centre for Biotechnology Information (NCBI) website: http://www.ncbi.nlm.nih.gov/blast/Blast.cgi. The following databases and tools were used to gain additional information on the BrpA protein: Conserved Domain Database (CDD), PROSITE motif search, SignalP 4.0, HMMER, TMHMM, HHPred, and Softberry BProm promoter search (www.softberry.com) [30]–[36].

The Fold and Functional Assignment System (FFAS03) is a profile-profile and fold recognition algorithm that can detect remote homology between proteins [37]. Profile-profile comparisons have increased sensitivity compared to sequence-sequence or profile-sequence algorithms. FFAS03 searches numerous databases including non-redundant (nr) NCBI, Global Ocean Sampling (GOS) from JCVI, PDB, SCOP, and COG, as well as numerous metagenome datasets including MetaHit [11] which contains over 3 million unique genes from the human gut microbiome. The BrpA protein sequence was submitted to the server to identify proteins with homology based on FFAS profiling or sequence homology by BLAST and PSI-BLAST against the databases and metagenome datasets. The FFAS03 server can be found at: http://ffas.burnham.org/ffas-cgi/cgi/document.pl.

The Integrated Microbial Genomes and Metagenomes (IMG/M) [38] is a data management system for the comparative analysis of metagenome sequence data. IMG/M-HMP [39] specifically contains metagenome data from the Human Microbiome Project (HMP) [40]. It contains 748 metagenome datasets generated from sequencing samples from different body sites and also, tools for comparative analysis between hosted sequences and user supplied sequences. The BrpA protein sequence was used a query sequence to BLAST (1e-^05^ and 1e-^50^ maximum e-value cut-off) against all the available metagenomes from 17 body sites from the HMP dataset. The IMG/M-HMP server can be found at: http://www.hmpdacc-resources.org/cgi-bin/imgm_hmp/main.cgi.

### DNA manipulations and cloning

Induction of fosmids from LOW to high copy number was performed as per the manufacturer’s instructions. The Qiagen QIAprep Spin mini-prep kit was used to extract fosmids using the protocol outlined by manufacturer. The *brpA_L_, brpA_S_* and *brpAatfA* genes were amplified using ReddyMix PCR mastermix (Thermo Scientific). PCR products were purified with a Qiagen PCR purification kit and digested with restriction enzymes *XbaI* and *PstI* (Roche Applied Science), followed by ligation using the Fast-Link DNA ligase kit (Epicentre Biotechnologies) to similarly digested plasmid pCI372. Electro-competent *E. coli* MKH13 were transformed with the ligation mixture and plated on LB agar plates containing 20 µg/ml Cm for selection.

The pBAD TOPO TA expression kit (Invitrogen, Carlsbad CA, USA) was used to clone the PCR products into the pBAD expression vector according to the manufacturer’s instructions. The *brpA_L_*, *brpA_S_* and *brpAatfA* genes were amplified as outlined above. The resulting plasmids, containing the genes of interest were electroporated into freshly competent *E. coli* EPI300 and plated on LB agar containing 100 µg/ml of ampicillin.

Colony PCR was performed on resistant transformants using a gene and plasmid (pCI372 or pBAD) specific primer combination to confirm the presence and size of the insert. Inserts were sequenced to confirm the correct nucleotide sequence (GATC Biotech, Germany).

### Growth experiments

Cultures were grown overnight in the relevant media (LB or M9 broth). Cells were subsequently harvested, washed in one quarter strength sterile Ringer’s solution and re-suspended in fresh media. A 2% (v/v) inoculum was sub-cultured in fresh broth containing sodium chloride (NaCl), and 200 µl was transferred to a sterile 96-well micro-titer plate (Starstedt Inc. Newton, USA). For minimal media experiments, filter-sterilised stock solutions of the osmoprotectants betaine, L-carnitine and L-proline were added to a final concentration of 1 mM. Micro-titer plates were incubated at 37°C for 24–48 hours in an automated spectrophotometer (Tecan Genios) which recorded the OD 595nm every hour. The data was subsequently retrieved and analysed using the Magellan 3 software program.

Survival in high salt media in the presence and absence of 20 µM β-carotene was assessed by harvesting overnight cultures as above and sub-culturing in either 3% NaCl or 7% NaCl for MKH13 and EPI300 strains respectively. Cultures were incubated at 37°C both aerobically (with shaking) and anaerobically (static) for 48 hours. Subsequently, serial dilutions of cultures were made in one quarter strength sterile Ringers solution and plated on LB agar. Viable cells were enumerated and calculated as the number of colony forming units per millilitre (CFU/ml).

Graphs (created using SigmaPlot 10.0) are presented as the average of triplicate experiments, with error bars being representative of the standard error of the mean (SEM).

### Transposon mutagenesis

Transposon mutagenesis was carried out on SMG 6 using the EZTn-*5<*oriV/KAN-2> *in vitro* transposition kit (Epicentre Biotechnologies) in accordance with the manufacturer’s instructions. *E. coli* EPI300 cells were transformed with the transposon reaction mixture and selected on plates containing Cm and Kan (12.5 and 50 µg/ml, respectively). Transposon insertions in the regions of interest were confirmed by PCR. Regions containing the EZTn*5* transposon are approximately 1.9 kb larger than the region covered by the primers. PCR products of the correct size were sequenced from the ends of the transposon using the primers EZTn FP-1 and RP-1 (Table S1) to confirm the location of transposon insertion. All sequencing was performed by GATC Biotech (Germany).

## Results

### Screening the human gut metagenomic library

**Figure 1:**
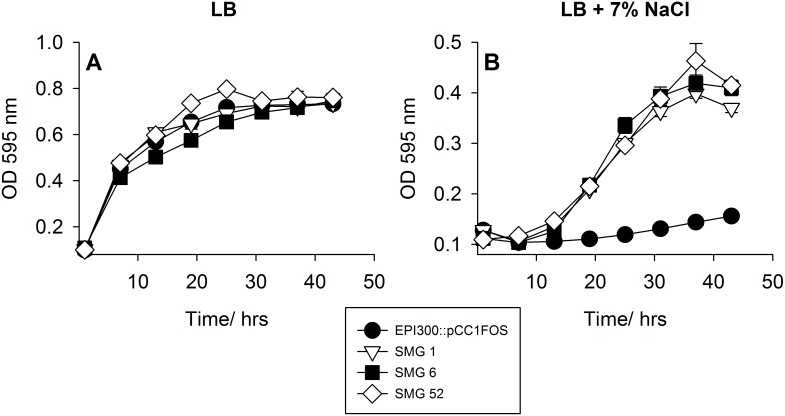
Growth of metagenomic clones SMG 1, 6 and 52 compared to EPI300 carrying an empty fosmid vector (pCC1FOS) in (A) LB broth and (B) LB broth supplemented with 7% NaCl.

Fifty-three salt-tolerant clones were identified from a screen of approximately 23,000 fosmid library clones. The clones were annotated as SMG (for Salt MetaGenome) 1–53. Six clones grew within 24 hours (SMG 1–6) and the remaining 47 grew over the following 24–48 hours. The focus of this study were clones SMG 1 and SMG 6, both of which were found to contain the same insert. SMG 6 was chosen for further analysis. Previous work has focused on clones SMG 3 and SMG 5 and SMG 25 [22], [23]. End sequencing revealed that another clone, SMG 52, shared the same sequences at the 5′ and 3′ ends of the fosmid as SMG 1 and SMG 6. Furthermore, SMG 52 displayed a similar growth profile to SMG 1 and 6 when grown under sodium chloride (NaCl) stress and all three clones have a significant (*P*<0.0001 for all clones) growth advantage in the presence of 7% added NaCl compared to the EPI300 host strain carrying the empty fosmid vector (pCC1FOS) (Figure 1B). No difference in growth between any of the clones was observed in LB alone (Figure 1A). Further investigation involving pyrosequencing revealed SMG 52 contained the same insert as SMG 1 and SMG 6.

### Fosmid sequencing and bioinformatic analysis

The fosmid inserts from SMG 1, 6 and 52 were fully sequenced and assembled by GATC Biotech (Germany) using the GS-FLX Titanium mini run. All three inserts were found to be identical, sharing 100% nucleotide identity over the entire length of the fosmid insert (∼34 kb). Gene prediction using FGENESB predicted the presence of thirty putative open reading frames (see Table 2). Translated nucleotide sequences were subjected to BLASTP (maximum e-value cut-off of 1e-^05^) analysis to identify homologous sequences in the database. The vast majority corresponded to proteins from the Gram-negative *Bacteroidetes* phylum, with amino acid identities ranging from 26% to 100%. Proteins with between 99%–100% amino acid identity corresponded to three species of *Bacteroides*, namely *Bacteroides thetaiotaomicron* VPI-5482, *Bacteroides sp.* 1_1_6 and *Bacteroides sp.* 1_1_14. The remainder corresponded to other members of the phylum *Bacteroidetes* from genera *Alistipes*, *Prevotella* and *Odoribacter*, as well to Gram-positive *Firmicutes* from the family *Lachnospiraceae* and genera *Clostridium* and *Veillonella*.

**Table 2:** List of putative proteins encoded on SMG 6 fosmid insert.

Gene #	Strand	Length (a.a)	BlastP top hit	Best hit organism (BlastP)	e-value	Query coverage	% ID (a.a)	% G+C
1	+	418	Hypothetical protein (BT_1366)	*Bacteroides thetaiotaomicron* VPI-5482	0.00E+00	100%	100%	48.05
2	–	124	Conserved hypothetical protein (DUF 3127)	*Bacteroides sp.* 1_1_6	6.00E−67	100%	100%	52.00
3	+	374	DNA polymerase III, chain beta (beta clamp superfamily)	*Bacteroides thetaiotaomicron* VPI-5482	0.00E+00	100%	100%	47.64
4	+	255	DNA polymerase III, epsilon chain (DDEHh exonuclease domain)	*Bacteroides sp.*1_1_6	4.00E−149	100%	100%	45.57
5	+	400	Phosphopantothenoylcysteine decarboxylase (Flavoprotein, DFP domains)	*Bacteroides sp.* 1_1_6	0.00E+00	100%	99%	49.38
6	+	555	DNA repair protein, RecN (ABC_RecN domains)	*Bacteroides sp.* 1_1_14	0.00E+00	100%	99%	50.66
7	+	247	tRNA/rRNA methyltransferase (SpoU_sub_bind and methylase domains)	*Bacteroides thetaiotaomicron* VPI-5482	5.00E−143	100%	100%	47.98
8	+	568	Tetratricopeptide repeat family protein (Trypsin_2 and TPR domains)	*Bacteroides sp.* 1_1_6	0.00E+00	100%	99%	46.34
9	+	190	Putative transcriptional regulator (NGN_SP_UpxY domain; NusG)	*Bacteroides thetaiotaomicron* VPI-5482	4.00E−135	100%	99%	42.76
10	+	122	Transcriptional regulator (UpxZ domain)	*Bacteroides sp.* 1_1_14	2.00E−63	100%	97%	42.82
11	+	789	Capsule polysaccharide export protein (Poly_export and SLBB domains)	*Bacteroides thetaiotaomicron* VPI-5482	0.00E+00	100%	99%	48.90
12	+	378	Uncharacterised protein, putative chain-length determining	*Bacteroides faecis* CAG:32	0.00E+00	100%	71%	42.83
13	+	647	Capsular polysaccharide biosynthesis protein (CapD) (UDP_invert_4–6DH_SDR_e domain)	*Bacteroides sp.* 1_1_6	0.00E+00	98%	87%	44.24
14	+	410	UDP-glucose dehydrogenase (NAD_Gly3P_dh_N and UDPG_MGDP_dh domains)	*Bacteroides fragilis* HMW 610	0.00E+00	100%	82%	43.23
15	+	348	NAD dependent epimerase/dehydratase (NADB_Rossmann superfamily)	*Bacteroides sp.* 4_1_36	3.00E−176	100%	84%	42.50
16	+	189	dTDP-4-dehydrorhamnose 3,5-epimerase (dTDP_sugar_isom domain)	*Bacteroides fragilis* CL07T00C01	3.00E−124	100%	90%	43.33
17	+	451	Alanine ABC transporter permease (DltB domain; MBOAT superfamily)	*Alistipes onderdonkii*	2.00E−136	97%	57%	37.20
18	+	292	Hypothetical protein Alfi_2997 (DUF 535 superfamily)	*Alistipes finegoldii* DSM 17242	1.00E−38	96%	30%	36.61
19	+	172	Hypothetical protein BF638R_1544 (RimL domain)	*Bacteroides fragilis* 638R	2.00E−67	99%	68%	39.35
20	+	368	Putative LPS biosynthesis transmembrane protein	*Bacteroides fragilis* 638R	4.00E−51	98%	36%	33.06
21	+	331	Hypothetical protein BSHG_0833	*Bacteroides sp.* 3_2_5	2.00E−83	100%	43%	31.02
22	+	479	Hypothetical protein, polysaccharide export (MATE_tuaB_like domain)	*Clostridium hathewayi* WAL-18680	6.00E−150	98%	46%	36.11
23	+	376	Hypothetical protein HMPREF9447_00823 (AHBA_syn domain; AAT_I superfamily)	*Bacteroides oleiciplenus* YIT 12058	0.00E+00	100%	81%	47.48
24	+	184	Putative acetyltransferase (LbetaH_MAT_like domain)	*Prevotella sp.* CAG:1092	6.00E−57	94%	55%	40.00
25	+	98	Hypothetical protein, putative acyltransferase	*Prevotella saccharolytica* F0055	7.00E−07	98%	37%	30.64
**26**	**+**	**271**	**Putative membrane protein**	***Prevotella sp.*** ** CAG:873**	**6.00E**−**10**	**99%**	**26%**	**32.05**
27	+	288	Hypothetical protein HMPREF0994_06876 (ATP-grasp_tupA domain)	*Lachnospiraceae bacterium* 3_1_57FAA_CT1	2.00E−81	99%	70%	35.01
28	+	366	Uncharacterised protein BN814_01473, putative glycosyltransferase (GT1_ams_like domain)	*Veillonella sp.* CAG:933	1.00E−132	98%	54%	39.69
29	+	366	Putative glycosyltransferase	*Bacteroides thetaiotaomicron* VPI-5482	2.00E−53	98%	37%	40.05%
30	+	364	Hypothetical protein HMPREF9449_00933	*Odoribacter laneus* YIT 12061	0.00E+00	98%	69%	40.43

**Abbreviations:** aa = amino acid; % ID = % identity at amino acid level over the entire length of the protein; % G+C = Percentage guanine and cytosine content;

DUF = Domain of Unknown Function.

Functional assignment of the encoded proteins on SMG 6 based on homology searches using BLASTP revealed that gene 26 was predicted to encode a putative membrane protein, although none of the potential homologues identified shared greater than 30% amino acid identity (placing them in the “twilight zone” of evolutionary relatedness). This protein also shared sequence similarity with a *brp/blh*-family 15,15′-β-carotene monooxygenase from *Prevotella marshii* DSM 16973 (28% identity over 254 amino acids) and with a proline symporter from *Bifidobacterium bifidum* BGN 4 (25% identity over 222 amino acids). Given that proline is an important osmoprotectant utilised by bacteria to counteract the deleterious effects of salt-induced osmotic stress [41], [42], we elected to pursue this gene, which we have named *brpA,* for further study.

### Features of SMG 6 and *brpA*/BrpA

**Figure 2:**
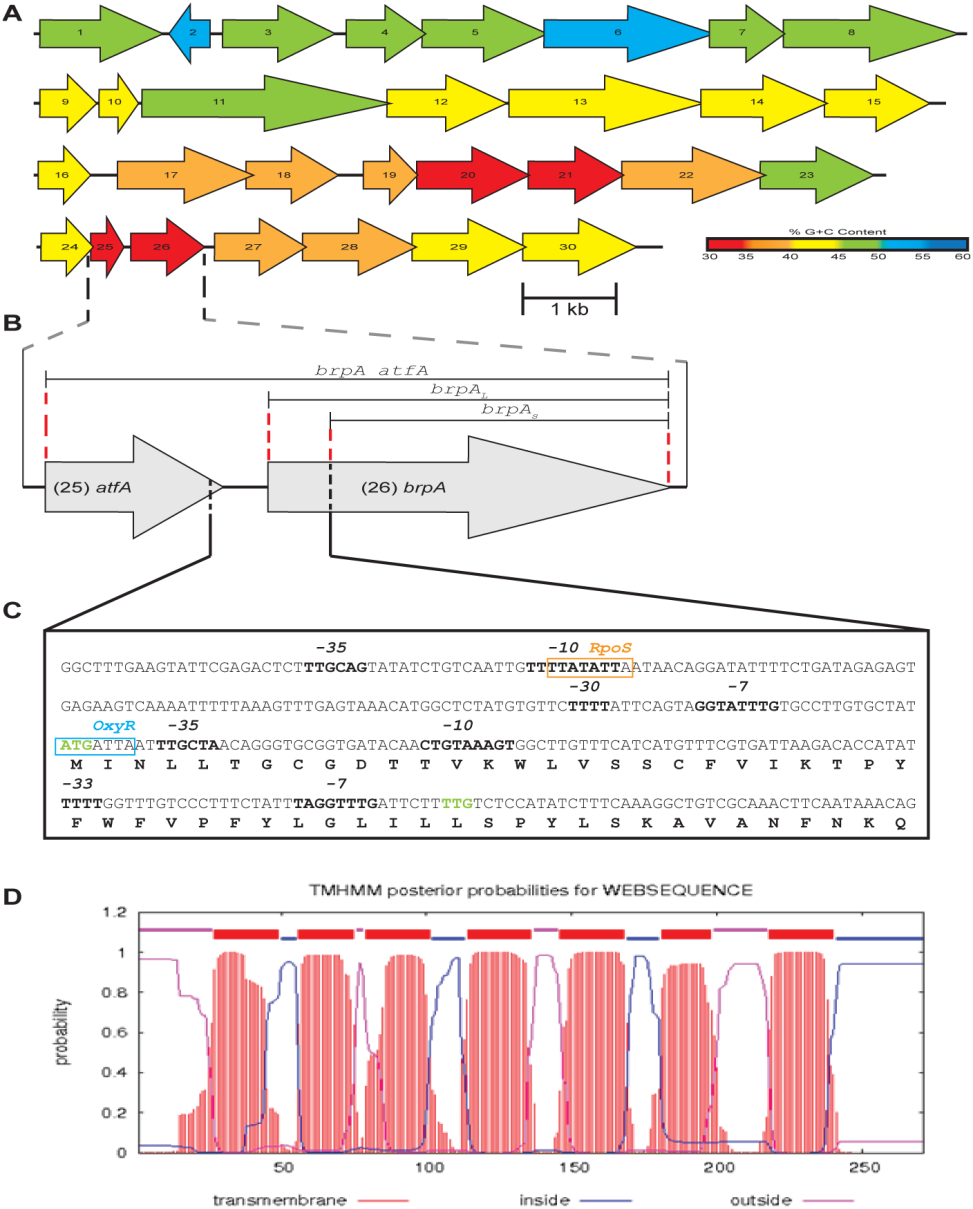
Overview of SMG 6 fosmid insert and features of specific genes. (**A**) Gene map of SMG 6 insert, displaying gene orientation and individual %G+C content indicated with a gradient colour bar. Gene numbers correspond to those in Table 2 and are drawn approximately to scale. (**B**) Focus on genes 25 (*atfA*) and 26 (*brpA*), showing the regions cloned for each construct. (**C**) Detailed view of putative ATG and TTG start codons of *brpA*, including upstream regions, as well as predicted promoter regions (highlighted in bold) and transcription factor binding site sequences (blue and orange boxes). (**D**) TMHMM prediction of seven transmembrane regions in BrpA.

The *brpA* gene is number 26 of the 30 predicted genes on SMG 6 (Fig. 2). It is predicted to be a lone open reading frame, preceded by and followed by a seven and a four gene operon, respectively. It is flanked upstream and downstream by a number of genes predicted to encode proteins with acetyl-, acyl- or glycosyl-transferase activities. There are indications that *brpA* and a number of adjacent genes have been acquired through lateral gene transfer (LGT). The SMG 6 fosmid insert is ∼34.26 kb and its overall %G+C content is 41.92%. The highest genetic identities of a large proportion of the genes are to *Bacteroides* species, with up to 100% identity in some cases. The %G+C content of genus *Bacteroides* ranges from 40–48%, with *B. thetaiotaomicron* VPI-5482, *Bacteroides sp.* 1_1_6 and *Bacteroides sp.* 1_1_14 all having a %G+C content of approximately 43% (Genomes Online Database, GOLD; http://www.genomesonline.org/). The %G+C content of the genes on the SMG 6 fosmid insert is illustrated in Figure 2A. Genes in the first half of the insert, up to and including gene 16, have a %G+C content of ∼45%; similar to the average %G+C content of the genus *Bacteroides*. The second half of the insert displays a clear drop in %G+C content to ∼37%. The %G+C content of some individual genes is also low, including *atfA* and *brpA* (Figure 2A), which share BLAST homology to low G+C Gram-positive bacteria, mainly from the Phylum *Firmicutes*.

The *brpA* gene was predicted to have different start codons using FGENESB depending on the settings used; the alternative start codon TTG (leucine) was predicted using “generic bacterial”, resulting in a 232 amino acid protein. Given that a number of the proteins on SMG 6 shared 100% amino acid identity with *Bacteroides thetaiotaomicron* VPI-5482, it was also chosen as the closest organism for gene prediction and predicted an ATG (methionine) as the start codon, 117 base-pairs upstream of the predicted TTG start codon (encoding a protein of 271 amino acids). GeneMark was used for gene prediction as a comparison and it also predicted the same ATG as the start codon. A putative ribosome binding site (RBS) sequence (AGGTTT) was found ending seven base-pairs upstream of TTG, while a stronger RBS sequence (AGTAGG) ended 19 base-pairs upstream of the ATG start codon. Putative *E.* coli-type −10 and −35 promoter regions were detected using BProm (www.softberry.com) upstream of both putative start codons. Manual inspection of upstream sequences also revealed the presence of a near perfect *Bacteroidetes −*7/−33 promoter region (TAGGTTTG/TTTT; consensus TAnnTTTG/TTTG) [43], [44] upstream of the TTG start codon and a GGTATTTG/TTTT at −14/−30 (GGTATTTG/TTTT) upstream of ATG. The predicted promoter sequences along with putative transcription factor binding sites can be seen in Figure 2C. A putative RpoS binding site is found upstream of the ATG start codon, while an OxyR binding sequence is predicted to be located upstream of the TTG start codon.

The BrpA protein was predicted to be a 30.9 kDa membrane protein with seven transmembrane regions as predicted with TMHMM (Figure 2D). BrpA has a predicted pI of 9.42 and is composed of ∼46% hydrophobic amino acids, similar to other microbial Brp/Blh proteins (pI range 8.89–9.56 and 48–56% hydrophobic amino acids) [24]. No signal peptide sequence, conserved domains or sequence motifs were detected for BrpA. We also searched for motifs in the protein sequences homologous to BrpA from BLAST. A lipocalin motif was detected in a hypothetical protein from *Clostridium sp* KLE-1755. Interestingly, lipocalin motifs are found in proteins that bind small hydrophobic molecules such as retinoids, carotenoids, lipids and steroids [45]. Table S2 shows the lipocalin motif and the corresponding motif identified in *Clostridium sp* KLE-1755. The BrpA amino acid sequence along with the top 10 BLAST homologues were aligned to identify conserved residues in these proteins. The residues that match the lipocalin motif are displayed in green and those that do not are in red (Table S2).

Due to low BLAST sequence identity, the FFAS03 server was used with the aim of identifying homologues to BrpA. The best homologues were an uncharacterised bacterial protein (COG 3274; acyltransferase) and a predicted membrane protein (COG 4763) with significant scores of −40.70 and −23.30 respectively. Interestingly the best hit homologue in the protein databank (PDB) was to an archaeal-type rhodopsin (3ug9), although the score of −9.43 did not reach significance (–9.50).

**Figure 3:**
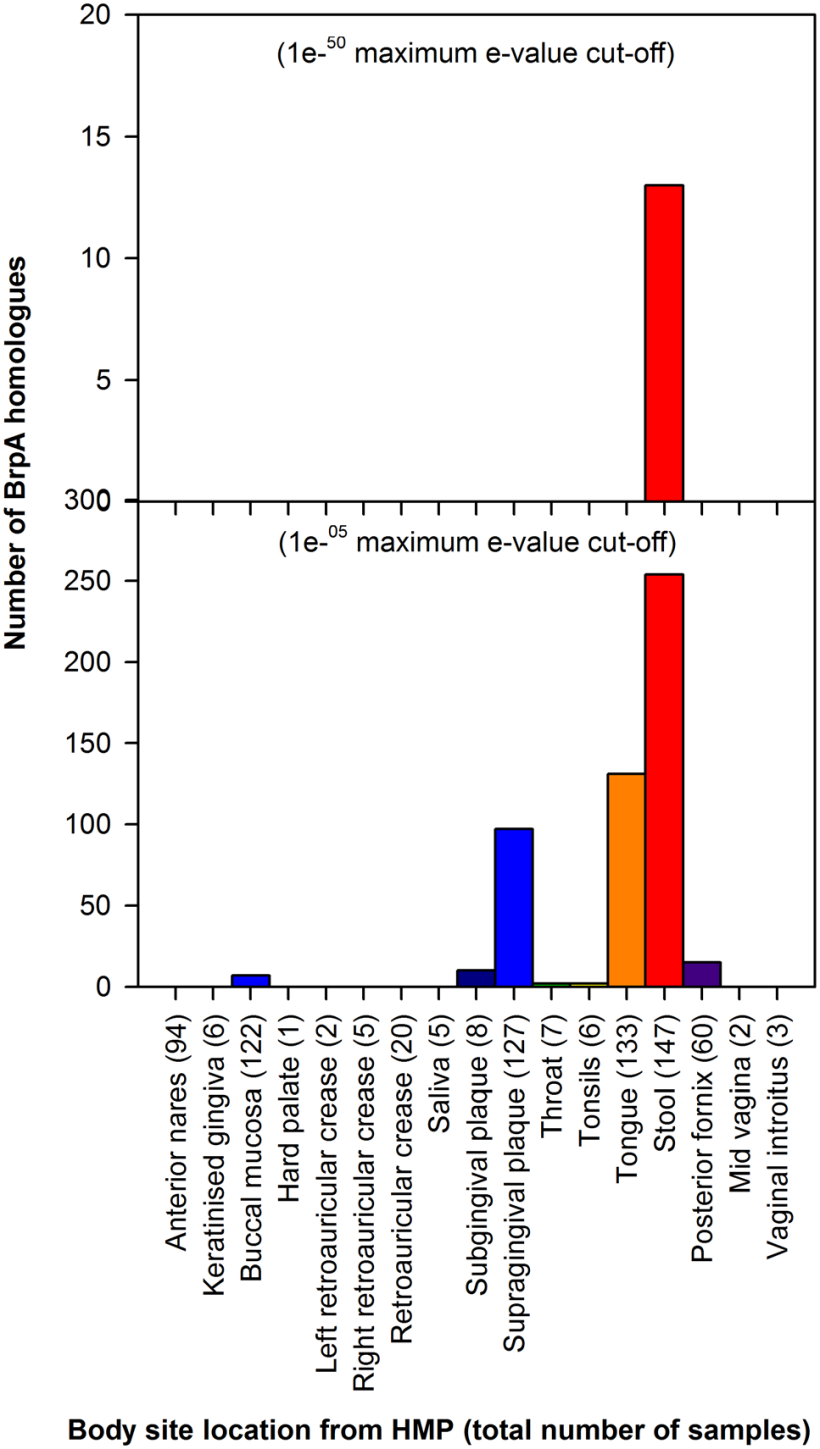
BrpA homologues identified when BLAST searched against Human Microbiome Project (HMP) datasets from 17 body sites at maximum e-value cut-off of (A) 1e^−50^ and (B) 1e^−05^.

The IMG/M-HMP database which contains all metagenomic datasets encompassing 17 body sites from the Human Microbiome Project (HMP) was also screened for BrpA homologues. Using a combination of the most lenient and strictest search criteria (maximum e-value cut-off of 1e-05 and 1e-50, respectively) BrpA homologues were identified in the HMP datasets (Figure 3). In addition, there were 145 hits to the MetaHit dataset using BLAST on the FFAS03 server.

### The *brpA* gene confers a salt tolerance phenotype when heterologously expressed in *Escherichia coli*

**Figure 4:**
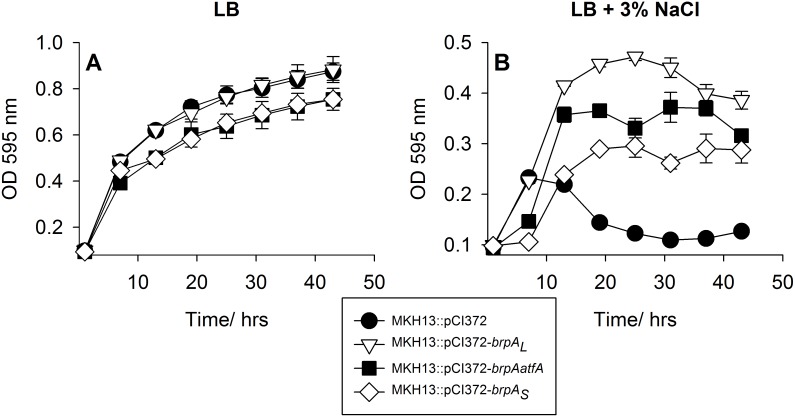
Growth of *E. coli* MKH13::pCI372 and *E. coli* MKH13 carrying a plasmid encoded copy of either *brpA_L_*, *brpA_S_* or *brpAatfA* in (A) LB broth or (B) LB+3% NaCl. All of the genes confer a significant salt tolerance phenotype to MKH13 relative to cells with an empty plasmid vector. All values are the average of triplicate experiments and error bars are representative of the standard error of the mean (SEM).

The *brpA* gene (gene 26) was cloned from both predicted start codons and expressed in *E. coli* MKH13. Both fragments increased the salt tolerance of MKH13 significantly. Cells expressing the larger fragment (*brpA_L_*) had the most significant effect (*P*  = 0.0002) in the presence of 3% NaCl. Although cells expressing the smaller fragment (*brpA_S_*) had a slower growth profile and a longer lag phase than the larger fragment (*brpA_L_*), both exhibited a significant growth advantage compared to the *E. coli* MKH13 control harbouring the empty plasmid (pCI372) (*P* = 0.0039) (Figure 4B). The gene immediately upstream of *brpA* is predicted to encode a 98 amino acid putative membrane protein (putative acyltransferase), which we have named *atfA*. The *atfA* gene was also cloned in combination with *brpA* (*brpAatfA*). Both genes in combination did not increase the salt tolerance of MKH13 relative to *brpA_L_* alone, when grown in LB+3% NaCl, but the increase in salt tolerance was significant (*P*  = 0.0002) (Figure 4B).

### L-proline did not increase salt tolerance further

Once we had shown that the *brpA* gene could confer a salt tolerance phenotype when expressed in *E. coli*, we aimed to decipher the mechanism of action and thereby assign a function to the encoded protein. Given that BLASTP analysis of the BrpA sequence revealed homology to a proline symporter, growth curves were carried out in minimal media supplemented with L-proline and also other common osmoprotectants, betaine and L-carnitine (final concentration of 1 mM). However, no growth advantage was seen in the presence of any of the added osmoprotectant compounds, suggesting that BrpA is not an osmoprotectant uptake system.

### Functional annotation of *brpA*

**Figure 5:**
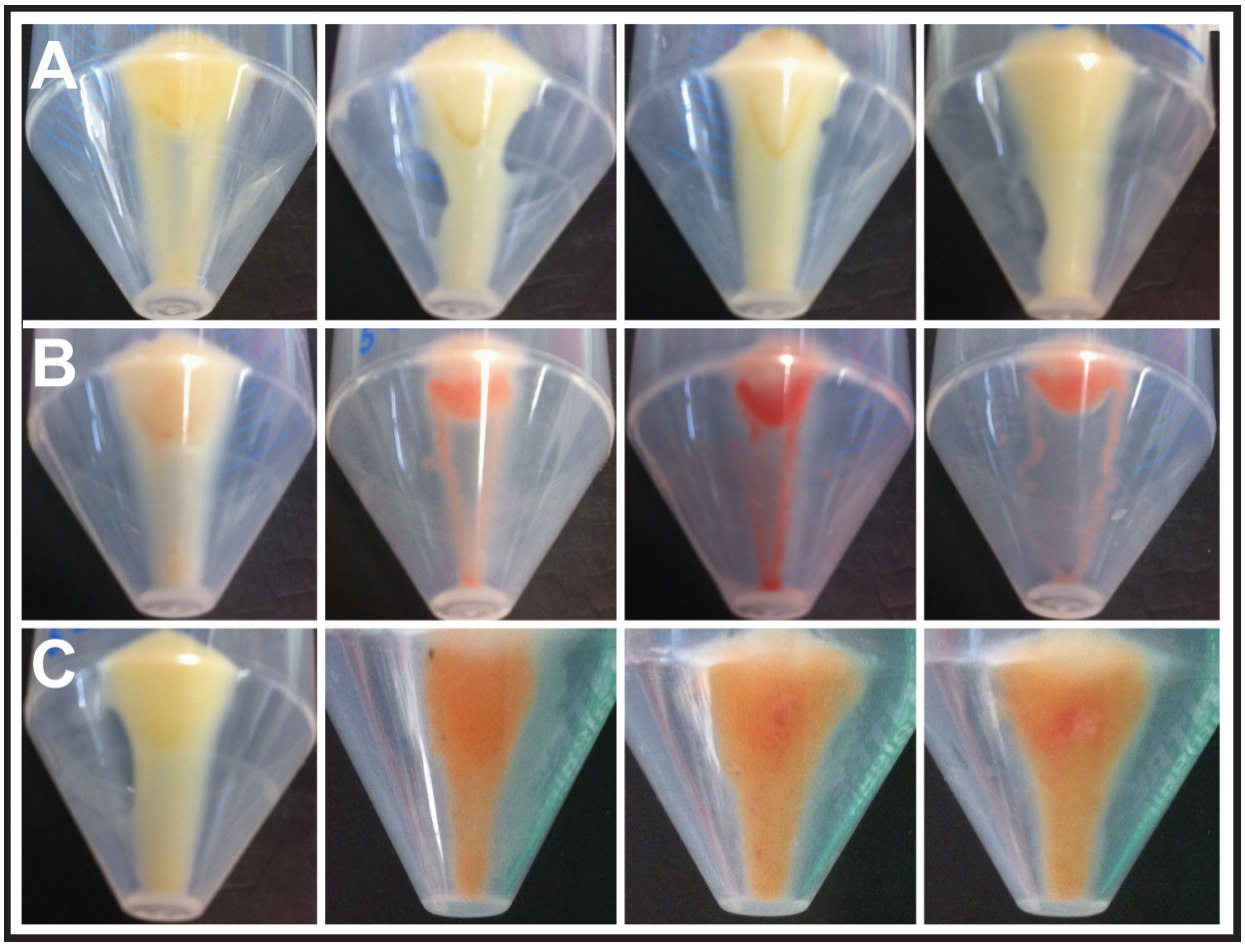
Pigmentation observed in cell pellets. (**A**) Appearance of cell pellets grown in LB supplemented with β-carotene. From left to right: *E. coli* MKH13::pCI372, MKH13::pCI372-*brpA_L_*, MKH13::pCI372-*brpA_S_* and MKH13::pCI372-*brpAatfA*. (**B**) Appearance of cell pellets of clones grown in LB supplemented with β-carotene and Copy Control Induction solution (L-arabinose). From left to right: *E. coli* EPI300::pCC1FOS, SMG 1, SMG 6 and SMG 52. (**C**) Appearance of cell pellets grown in LB supplemented with β-carotene and L-arabinose. From left to right: *E. coli* EPI300::pBAD, EPI300::pBAD-*brpA_S_*, EPI300::pBAD-*brpA_L_*, and EPI300::pBAD-*brpAatfA*.

BLASTP analysis also revealed that the BrpA protein exhibited homology to a *brp/blh*-family β-carotene 15,15′-monooxygenase. Such proteins are related to bacteriorhodopsins [24], and are annotated as bacterio-opsin related protein (brp)/brp-like homologue (blh) protein. Brp/Blh proteins have been shown to have β-carotene 15,15′-monooxygenase activity; cleaving β-carotene into two molecules of *all-trans* retinal (vitamin A aldehyde) [25]. The derived retinal is bound by a rhodopsin protein and cells expressing such proteins acquire an orange/red colour, indicative of the presence of retinal in the cell membrane [46]–[48]. Strains harbouring *brpA* were grown in the presence of β-carotene and cell pellets were observed for the development of the characteristic red/orange colour. *E. coli* MKH13 cells carrying the *brpA* gene on the pCI372 plasmid did not show any obvious colour development, most likely due to the fact that pCI372 is not inducible (Figure 5A).

Given that a number of previous studies have reported a requirement for the use of an inducible vector to visualise pigmentation in cell pellets [46]–[50], we cultured the original fosmid clones (which can be induced due to Copy Control capability of pCC1FOS fosmid vector) in the presence of β-carotene and included an induction solution to induce the fosmid from low to high-copy number. The cell pellets developed an intense red/orange colour while cells with an empty vector did not (Figure 5B). To confirm that the BrpA protein was responsible for this phenotype, we cloned *brpA* in isolation into the pBAD inducible expression vector and transformed it into *E. coli* EPI300 and repeated the growth experiments. Again, the cell pellets developed a distinctive a red/orange colour (Figure 5C).

### *brpA* also confers salt tolerance to *E. coli* EPI300

The genes (*brpA_L_, brpA_S_ and brpAatfA*) were also cloned into the pBAD expression vector and transformed into *E. coli* EPI300. All of the transformed strains exhibited increased salt tolerance relative to the host containing the empty pBAD vector, although EPI300::pBAD-*brpA_S_* to a lesser extent, similar to our observations with MKH13 above (Figure S1).

### Effect of β-carotene on survival in high-salt media

The effect of β-carotene on survival of both *E. coli* MKH13 and EPI300 strains was assessed. Survival of strains carrying a plasmid-encoded copy of *brpA* was compared to controls (carrying an empty plasmid) in high-salt media (3% NaCl for MKH13 and 7% for EPI300) in the presence and absence of β-carotene after a 48-hour period, both aerobically and anaerobically (Figure S2). Β-carotene did not provide an osmoprotective effect during salt stress to control strains or strains carrying a copy of the *brpA* gene under the conditions tested, however an increased salt tolerance phenotype was observed under both aerobic and anaerobic conditions.

### Transposon mutagenesis

**Figure 6:**
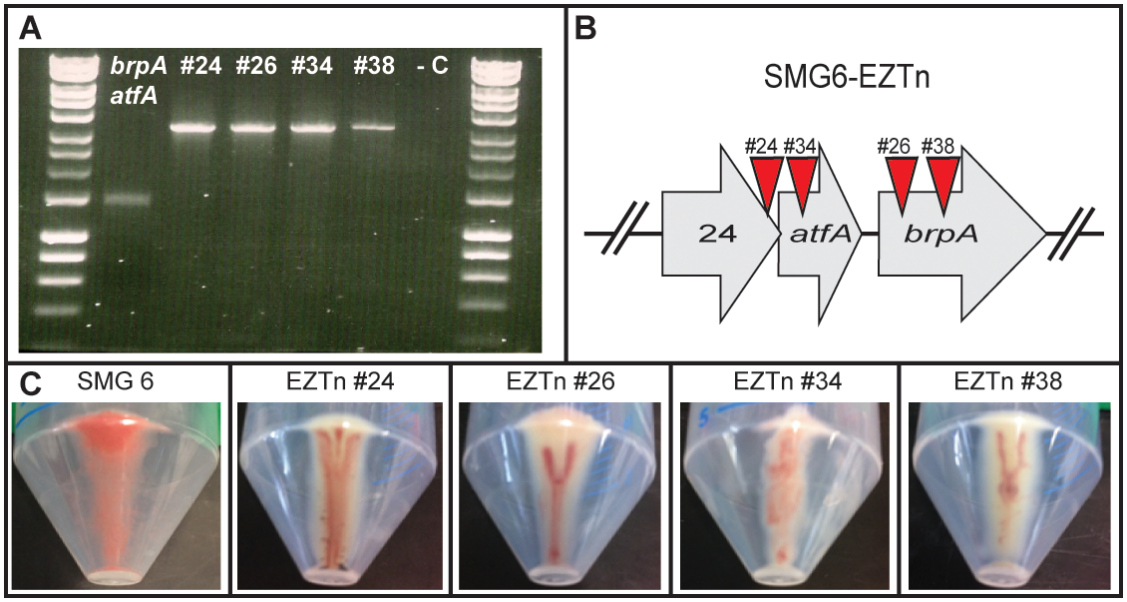
EZTn*5* transposon mutagenesis of SMG 6 was performed to identify mutants lacking pigmentation when grown in the presence of β-carotene. (**A**) Clones positive for a transposon in this region of SMG 6 fosmid insert were identified by PCR, with amplicons of ∼3.3 kb indicative of an insertion event. (**B**) Approximate locations of transposon insertions in relation to *brpA* and neighbouring genes. **(C)** Appearance of cell pellets of SMG 6 and transposon insertion mutants (EZTn #24, #26, #34 and #38) following growth in the presence of β-carotene.

Transposon mutagenesis was performed using the EZTn*5 in vitro* transposition system (Epicentre Biotechnologies) to create knock-out mutants of SMG 6. Clones harbouring a transposon insertion in the *brpA* and neighbouring genes were identified by PCR. The primer pair *brpAatfA* FP and RP were used to amplify this region, generating PCR products of ∼1.4 kb in the absence of a transposon insertion and products of ∼3.3 kb if the transposon was present (Figure 6A). Once positive clones were identified, the location of the transposon was confirmed by sequencing from the ends of the transposon. We identified four transposon mutants in SMG 6; namely 6-EZTn #24, #26, #34 and #38. The location of the transposon insertions are presented in Figure 6B. The aim was to identify clones that lacked pigmentation following transposition. Clones containing a transposon insertion do not display the same intense red pigmentation seen with SMG 6 and although there is visibly less pigmentation, some residual colour nevertheless remains (Figure 6C).

## Discussion

In the current study we have identified and characterised a novel salt tolerance locus from the human gut microbiome. Functional assignment of its encoded protein, BrpA, using BLAST returned homologues mainly annotated as hypothetical or putative membrane proteins. The only clue to the possible function of the protein was that it also shared sequence similarity (albeit at <30%) to a proline symporter and a *brp/blh*-family β-carotene 15,15′-monooxygenase. Sequence homologies of less than 30% are considered to be in the “twilight zone” and confidence of functional annotations diminishes below this threshold [51], [52]. Nevertheless, we felt it was worth investigating this gene further as proline is a well-known compound utilised by bacteria as an osmoprotectant when exposed to osmotic stress.

**Figure 7:**
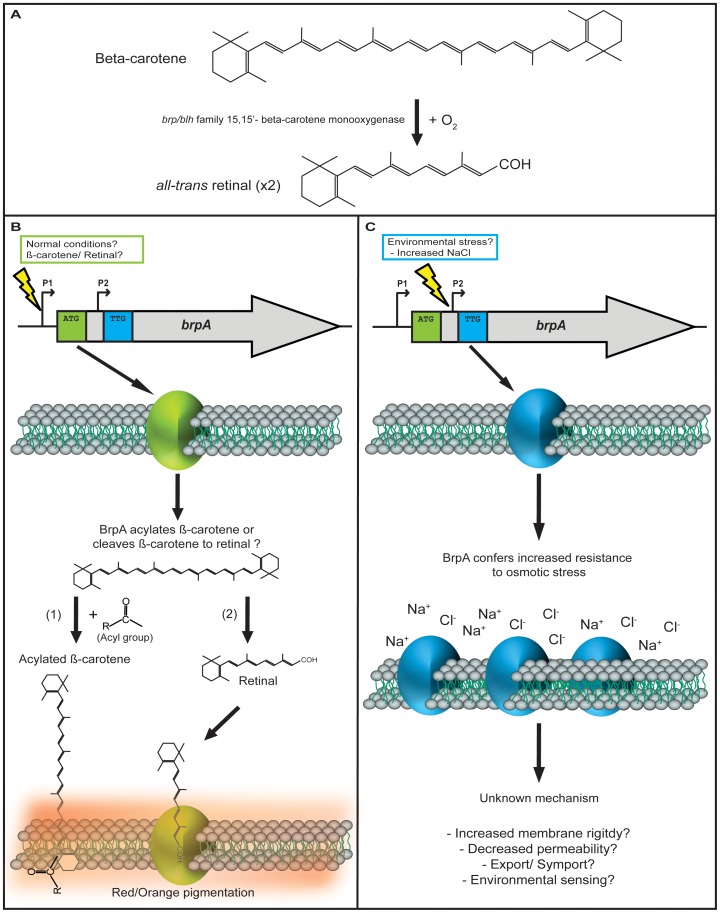
Possible mechanism(s) of action of BrpA (A) Representation of the known reaction for the formation of retinal. B-carotene is cleaved at its central 15,15′ bond by *brp* 15,15′- β-carotene monooxygenase to form two molecules of *­all-trans* retinal (Vitamin A aldehyde). We propose that *brpA* may be regulated from two promoters, with translation being initiated from one of two potential start codons (ATG and TTG), depending on environmental conditions. While speculative, we illustrate some possibilities discussed in the text. (**B**) Pigmentation phenotype: regulation of *brpA* from promoter 1 (upstream of ATG start codon) under “normal” cellular conditions, or possibly by β-carotene, could result in (**B1**) BrpA adding an acyl group to β-carotene, allowing it to interact with phosphate head groups of lipids and anchoring it in the hydrophobic core of the lipid membrane or (**B2**) BrpA may cleave β-carotene to retinal and subsequently bind the derived retinal anchoring it in the cell membrane. (**C**) Stress response: regulation of *brpA* from promoter 2 (upstream of TTG start codon), may be initiated by environmental signals such as changes in external osmolarity, resulting in increased tolerance or resistance to environmental stress, such as increased NaCl concentrations by an as yet unknown mechanism. Alternative start codons, such as TTG, have been found in a number of stress response genes.

Growth experiments in minimal media supplemented with L-proline and other osmoprotectants had no effect on growth or salt tolerance. The gene, which we have termed *brpA*, possibly encodes a putative *brp/blh*-family β-carotene 15,15′-monooxygenase. Such proteins have been shown to catalyse the conversion of β-carotene into two molecules of *all-trans* retinal (vitamin A aldehyde) (Figure 7A) [24], [25]. Growth of the metagenomic clone SMG 6 in the presence of exogenous β-carotene resulted in the cell pellets with a distinctive orange/red colour. A number of other studies have shown that bacterial cells expressing plasmid encoded β-carotene biosynthesis genes in addition to a *brp/blh* gene and a proteorhodopsin (PR) encoding gene adopt a similar colour due to the cleavage of β-carotene to retinal and subsequent binding of retinal by proteorhodopsins in the cell membrane [46]–[49]. The absence of any obvious PR encoding gene on SMG 6 therefore, does not explain the presence of colour in the SMG clones’ cell pellet. Furthermore, when *bprA* was cloned in isolation the cell pellets still had pigmentation, indicating that *brpA* alone is sufficient to confer this phenotype. There are however a few possible explanations for the pigmentation; *in silico* analysis reveals that *brpA* is predicted to have acyltransferase activity (COG 3274), as is *atfA*, the gene immediately upstream of *brpA*. The *atfA* gene was cloned in combination with *brpA*, however expression of both genes together had no appreciable effect on the degree of pigmentation or salt tolerance observed. Carotenoids and retinoids are hydrophobic, lipophilic molecules. The majority of carotenoids are found embedded in the hydrophobic core of lipid membranes and in lipid globules and other hydrophobic environments [53], [54]. Acylated carotenoids have been shown to be inserted in the membrane and the predicted acyltransferase activity of BrpA may explain the cell pellet pigmentation in the absence of a rhodopsin protein [55]. In *Staphylococcus aureus*, an acyltransferase is a key enzyme in the biosynthesis pathway for the orange carotenoid staphyloxanthin [56]. This enzyme was initially thought to carry out the final step in staphyloxanthin biosynthesis, although more recently it has been shown that it is actually the penultimate step [57]. The transfer of a polar acyl group or acyl-containing groups such as hydroxyl or keto groups to carotenoids would be likely to enable their interaction with phosphate head groups of lipids, thus anchoring them within membranes [55], [58].

The presence of a lipocalin motif was identified in a BLAST homologue of BrpA. Lipocalin proteins can bind hydrophobic molecules such as carotenoids and retinoids. It seems unlikely however, that this is the case with BrpA since the motif is quite different and lacks the characteristic glycine-X-tryptophan (G-X-W) signature found in almost all lipocalins [59]. The BrpA protein has seven predicted transmembrane regions, a characteristic shared with rhodopsin proteins [60]. It has previously been suggested that Brp/Blh-like proteins may be multifunctional and both cleave β-carotene and subsequently transport or bind the derived *all-trans* retinal, although this has not been demonstrated experimentally [25].

Four transposon mutants of SMG 6 were identified in this study using PCR. It was expected to obtain mutants that lack pigmentation when grown in the presence of β-carotene. While there is a clear visible difference in the appearance of the cell pellets of the mutants compared to SMG 6, each of the mutants retain some level of pigmentation, albeit to a lesser degree and with diminished colour intensity. Transposon insertion in genes upstream of *brpA* (mutants #24 and #34) indicates a polar effect mediating the reduction in the degree of pigmentation. It is surprising that some pigmentation remains in clones containing a transposon within the *brpA* gene (mutants #26 and #38), indicating residual carotenoid accumulation, possibly due to acyltransferase activity of *atfA*.

The % G+C content of individual genes on SMG 6 drops as low as 30.64% for gene 25 (*atfA*), while its neighbouring gene, *brpA*, is 32.05%. In addition, only 12% of the top 100 BLASTP hits to BrpA are predicted to be from Gram-negative bacteria. The remaining 88% are represented in the main by proteins with similarity to the low G+C, Gram-positive *Firmicutes* phylum, mainly from the genera of *Clostridium, Enterococcus* and *Streptococcus* among others. Taken together, these observations suggest much of this region, including the especially low % G+C, *atfA* and *brpA* genes, were acquired through a LGT event [61], [62]. Indeed, in support of this there is evidence that *brp*/*blh*-type genes, along with rhodopsins, undergo frequent LGT events [46], [63]–[65]. In β-carotene producing bacteria, only these two genes are required to produce retinal which is bound to the rhodopsin protein giving the recipient bacterium the ability to harvest light energy non-photosynthetically and convert it to chemical energy. Acquiring a rhodopsin gene in the gut would be somewhat redundant owning to the aphotic nature of the gut environment. A *brp/blh* β-carotene monooxygenase however could be beneficial to break down dietary-derived β-carotene.

There were two possible start codons predicted for the *brpA* gene using the FGENESB gene prediction program. Using the “bacterial generic” parameter as closest organism, a gene (*brpA_S_*) encoding a 232 amino acid protein with the alternative initiation codon TTG (leucine) was predicted. Because a number of proteins encoded on SMG 6 shared 100% amino acid identity with *Bacteroides thetaiotaomicron* VPI-5482, this organism was also used as the “closest organism” parameter. Using *B. thetaiotaomicron* VPI-5482 as “closest organism” predicted a gene (*brpA_L_*) encoding a 271 amino acid protein with an ATG (methionine) start codon. GeneMark also predicted ATG to be the start codon. Cloning and expression of the gene from both predicted start codons conferred salt tolerance to *E. coli*, although strains expressing the *brpA_L_* fragment had a shorter lag phase and reached a higher final OD. Initially, it seemed likely that ATG was the true start codon of *brpA*, however further manual inspection of the sequences upstream of both start codons revealed a characteristic *Bacteroides −*7/−33 promoter region preceding the TTG codon that deviated from the consensus by only one nucleotide. There is also a potential *Bacteroides-*type promoter upstream of ATG, but at position −14/−30 (GGTATTTG/TTTT). It therefore seems likely that TTG is the actual start codon in *Bacteroides*. Interestingly, previous studies have shown that the use of alternative initiation codons, other than ATG, is a common feature of osmotolerance genes in a number of gastrointestinal pathogens [12], [17]. The increased salt tolerance phenotype of *brpA_L_* compared to *brpA_S_* may be due to the fact that ATG is the most commonly utilised codon to initiate translation (∼90% of genes) in *E. coli* [66] and also the presence of strong RBS (AGUAGGU) upstream of the ATG start codon, which differs from the *E. coli* consensus RBS (AGGAGGU) by only one nucleotide. Taken together, the ATG start codon and strong *E. coli* RBS likely gives rise to more efficient levels of transcription and translation, as well as increased expression of *brpA* in *E. coli*, at least under the conditions tested in the current study. It is of course possible that the two protein types (long and short) are expressed under different environmental conditions, as was previously reported for the multi-stress resistance locus HtrA [67].

The presence of a putative RpoS binding site is predicted upstream of the ATG start codon of *brpA*. The alternative sigma factor (sigma 38) RpoS is the master regulator of the general stress response induced during stationary phase in *E. coli* and other Gram-negative bacteria [68]. In addition RpoS regulates the expression of a large number of genes in response to various stresses, including salt stress [69]–[71]. There is also a putative OxyR binding site in the upstream region of *brpA*. OxyR is a regulator of the oxidative stress response in many bacteria [72] and carotenoids can function as anti-oxidants and can increase resistance to oxidative stress [73], [74]. It is possible that the *brpA* gene is transcribed from two promoters under different environmental conditions, similar to the type of regulation seen with the osmoprotectant transporter ProP in *E. coli,* where the *proP* gene is transcribed from promoter 1 (P1) primarily in response to changes in osmolarity and from promoter 2 (P2) during stationary phase [75], [76].

The BrpA amino acid sequence was used to BLAST search against all metagenomes from the HMP dataset at the lowest (1e-^05^) and highest (1e-^50^) e-value. Hits to BrpA were most abundant in the stool, supra-gingival plaque and tongue metagenome samples at the lowest e-value (Figure 3B). The majority of these hits had quite low percentage identities in the range of 25%–35%. When the e-value cut-off was increased to 1e-^50^ only 13 putative BrpA homologues were identified and only from the stool metagenome samples (Figure 3A) and would therefore appear to be a rare gene found in some strains of *Bacteroides thetaiotaomicron*, which is one of the most abundant species in the human gut microbiome, having been shown to comprise 6% of all bacteria among the human gut microbiota [77]. It is interesting that homologues of this gene are found most abundantly in body sites (tongue, sub- and supragingival plaque and gut lumen/stool) where the microbiota would encounter β-carotene (i.e. from dietary sources).

Carotenoids have been shown to protect cells from various environmental stresses such as osmotic, oxidative and light as well as reinforcing and providing increased membrane rigidity [54], [74], [78]–[80]. In this study, β-carotene however did not provide any further increase in salt tolerance under the conditions tested and therefore does not appear to function in an osmoprotective capacity. Acyltransferase enzymes have also been linked to various stress responses, including osmotic stress. For example, the acyltransferase HtrB, provides protection against and exhibits increased expression in response to heat, acid, oxidative and osmotic stress in *Campylobacter jejuni* and *Salmonella typhimurium* [81], while acyltransferases have also been linked to the stress response in *Pseudomonas putida* [82].

In the current study we have used a combined functional metagenomic and bioinformatic approach to identify a novel gene from the human gut microbiome that has not previously been linked to salt tolerance. The gene, *brpA*, encodes a protein with homology to a *brp/blh*-family β-carotene 15,15′-monooxygenase. When expressed in *E. coli*, BrpA confers salt tolerance phenotype and cell pellets adopt a red/orange pigmentation when grown in the presence of exogenous β-carotene.

## Supporting Information

Figure S1 **Growth of** ***E. coli*** **EPI300::pBAD and EPI300::pBAD-** ***brpA_S_*** **(** ***P*** **= 0.0008), EPI300::pBAD-** ***brpA_L_*** **(** ***P*** **= 0.0002) and EPI300::pBAD-** ***brpAatfA*** **(** ***P*** **= 0.0001) in (A) LB broth and (B) LB broth supplemented with 7% NaCl.** All three strains had a statistically significant increased salt tolerance compared to EPI300 carrying an empty copy of the pBAD vector. Numbers in parentheses indicate significant *P* values (unpaired student *t*-test). All values are the average of triplicate experiments and error bars are representative of the standard error of the mean (SEM).
(PDF)

Figure S2 **The effect of β-carotene on the survival of MKH13 strains and EPI300 strains was assessed under aerobic and anaerobic conditions in (A) LB broth with 3% NaCl and (B) LB broth with 7% NaCl.** Viable cells were determined by calculating the average CFU per millilitre after 48 hours. Results are representative of triplicate experiments and error bars are the standard error of the mean (SEM).
(PDF)

Table S1 **^a^Restriction enzyme cut-sites are underlined (** ***PstI,*** **CTGCAG;** ***XbaI,*** **TCTAGA).**
(PDF)

Table S2 **^1^strain 3_1_57FAA_CT1.** A lipocalin motif was found in a homologue of BrpA from *Clostridium sp.* KLE1755. Lipocalin proteins can bind hydrophobic molecules such as carotenoids and retinoids. The top ten BLASTP homologues to BrpA were aligned to compare these protein sequences and identify putative lipocalin motifs. The consensus motif is displayed on the top row of Table 4. Residues that match the consensus are shown in green and mismatches are shown in red.
(PDF)
