# Supplementary material for: Metagenomic Identification of a Novel Salt Tolerance Gene from the Human Gut Microbiome Which Encodes a Membrane Protein with Homology to a brp/blh-Family β-Carotene 15,15′-Monooxygenase
Source: PLoS One. 2014 Jul 24;9(7):e103318. doi: 10.1371/journal.pone.0103318 (PMC4110020; doi:10.1371/journal.pone.0103318)
Supplement: Figure S1 — Growth of E. coli EPI300::pBAD and EPI300::pBAD- brpAS ( P = 0.0008), EPI300::pBAD- brpAL ( P = 0.0002) and EPI300::pBAD- brpAatfA ( P = 0.0001) in (A) LB broth and (B) LB broth supplemented with 7% NaCl. All three strains had a statistically significant increased salt tolerance compared to EPI300 carrying an empty copy of the pBAD vector. Numbers in parentheses indicate significant P values (unpaired student t-test). All values are the average of triplicate experiments and error bars are representative of the standard error of the mean (SEM). (PDF) [file pone.0103318.s001.pdf]

Figure S1. Growth in LB and LB + 7% NaCl

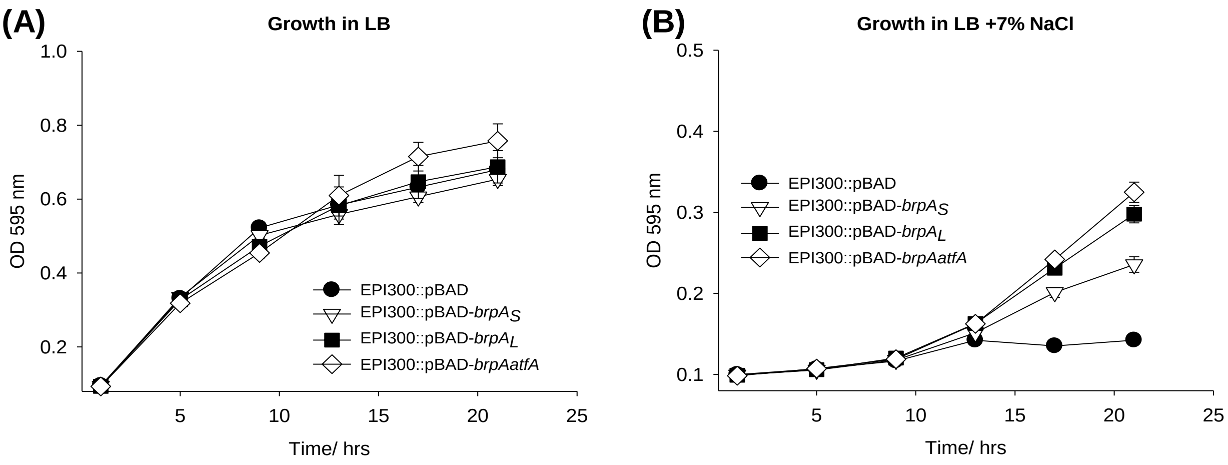

Figure S1. Growth of *E. coli* EPI300::pBAD and EPI300::pBAD-*brpA*$_S$ (P = 0.0008), EPI300::pBAD-*brpA*$_L$ (P = 0.0002) and EPI300::pBAD-*brpAatfA* (P = 0.0001) in **(A)** LB broth and **(B)** LB broth supplemented with 7% NaCl. All three strains had a statistically significant increased salt tolerance compared to EPI300 carrying an empty copy of the pBAD vector. Numbers in parentheses indicate significant *P* values (unpaired student t-test). All values are the average of triplicate experiments and error bars are representative of the standard error of the mean (SEM).
