# Supplementary material for: Metagenomic Identification of a Novel Salt Tolerance Gene from the Human Gut Microbiome Which Encodes a Membrane Protein with Homology to a brp/blh-Family β-Carotene 15,15′-Monooxygenase
Source: PLoS One. 2014 Jul 24;9(7):e103318. doi: 10.1371/journal.pone.0103318 (PMC4110020; doi:10.1371/journal.pone.0103318)
Supplement: Figure S2 — The effect of β-carotene on the survival of MKH13 strains and EPI300 strains was assessed under aerobic and anaerobic conditions in (A) LB broth with 3% NaCl and (B) LB broth with 7% NaCl. Viable cells were determined by calculating the average CFU per millilitre after 48 hours. Results are representative of triplicate experiments and error bars are the standard error of the mean (SEM). (PDF) [file pone.0103318.s002.pdf]

Figure S2.

(A)

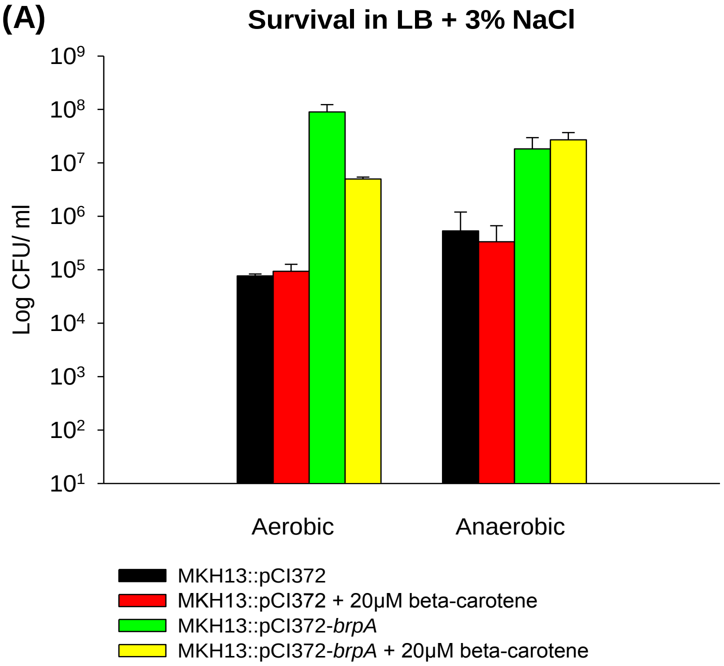

(B)

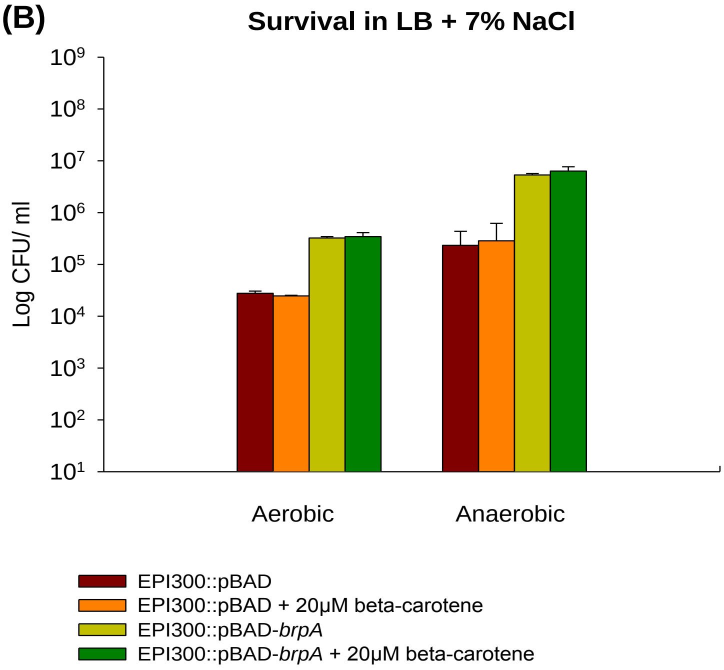

Figure S2. The effect of beta-carotene on the survival of MKH13 strains and EPI300 strains was assessed under aerobic and anaerobic conditions in **(A)** LB broth with 3% NaCl and **(B)** LB broth with 7% NaCl. Viable cells were determined by calculating

the average CFU per millilitre after 48 hours. Results are representative of triplicate experiments and error bars are the standard error of the mean (SEM).
