# Supplementary material for: Metagenomic Identification of a Novel Salt Tolerance Gene from the Human Gut Microbiome Which Encodes a Membrane Protein with Homology to a brp/blh-Family β-Carotene 15,15′-Monooxygenase
Source: PLoS One. 2014 Jul 24;9(7):e103318. doi: 10.1371/journal.pone.0103318 (PMC4110020; doi:10.1371/journal.pone.0103318)
Supplement: Table S1 — aRestriction enzyme cut-sites are underlined ( PstI, CTGCAG; XbaI, TCTAGA). (PDF) [file pone.0103318.s003.pdf]

Table S1. Oligonucleotide primers

**Primer**	**Sequence (5' – 3')[a]**
pCI372 FP	CGGGAAGCTAGAGTAAGTAG
pCI372 RP	CCTCTCGGTTATGAGTTAG
pBAD FP	ATGCCATAGCATTTTTATC
pBAD RP	GATTTAATCTGTATCAGG
brpA$_S$ FP	AAAACTGCAGACCCAACACGATGCCATATT
brpA$_S$ RP	GCTCTAGATAACAGGGTGCGGTGATACA
brpAatfA FP	AAAACTGCAGTAGCGGCTGGATCGGTAGTA
brpAatfA RP	GCTCTAGAACCCAACACGATGCCATATT
brpA$_L$ FP	AAAACTGCAGGCCGAATATCAACCCAACAC
brpA$_L$ RP	GCTCTAGAAGGTATTTGTGCCTTGTGCT
EZTn-FP-1	GCCAACGACTACGCACTAGCCAAC
EZTn-RP-1	GAGCCAATATGCGAGAACACCCGAGAA

[a]Restriction enzyme cut-sites are underlined (*PstI,* CTGCAG; *XbaI,* TCTAGA)
