# Supplementary material for: Metagenomic Identification of a Novel Salt Tolerance Gene from the Human Gut Microbiome Which Encodes a Membrane Protein with Homology to a brp/blh-Family β-Carotene 15,15′-Monooxygenase
Source: PLoS One. 2014 Jul 24;9(7):e103318. doi: 10.1371/journal.pone.0103318 (PMC4110020; doi:10.1371/journal.pone.0103318)
Supplement: Table S2 — 1strain 3_1_57FAA_CT1. A lipocalin motif was found in a homologue of BrpA from Clostridium sp. KLE1755. Lipocalin proteins can bind hydrophobic molecules such as carotenoids and retinoids. The top ten BLASTP homologues to BrpA were aligned to compare these protein sequences and identify putative lipocalin motifs. The consensus motif is displayed on the top row of Table 4. Residues that match the consensus are shown in green and mismatches are shown in red. (PDF) [file pone.0103318.s004.pdf]

Table S2. Putative lipocalin motifs in BrpA and its homologues

	LIPOCALIN MOTIF	[DENG]	{A}	[DENQG STARK]	X (0,2)	[DENQ ARK]	[UVFY]	{CP}	G	{C}	W	[FYWLRH]	{D}	[LIVMTA]
***Clostridium sp.* KLE 1755**	NPSRLAGAWYLVP	N	P	S	-	R	L	A	G	A	W	Y	L	V
**BrpA SMG 6**	LFSSMRDSIYLIPS	L	F	S	-	S	M	R	D	S	I	Y	L	I
***Prevotella sp.* CAG:873**	DVHGALHSWWFVP	D	V	H	-	G	A	L	H	S	W	W	F	V
***Prevotella buccalis* ATCC 35310**	VWQGMLDDSLFMF	V	W	Q	-	G	M	L	D	D	S	L	F	M
***Prevotella sp.* CAG:279**	DVHSWLHSWAFVP	D	V	H	-	S	W	L	H	S	W	A	F	V
***Prevotella marshii* DSM 16973**	PQTDFITSWSFLP	P	Q	T	-	D	F	I	T	S	W	S	F	L
***Lachnospiraceae bacterium*[1]**	NPSQMADKWYLVP	N	P	S	-	Q	M	A	D	K	W	Y	L	V
***Prevotella saccharolytica* F0055**	PQTDFITSWSFLP	P	Q	T	-	D	F	I	T	S	W	S	F	L
***Clostridium nexile* CAG:348**	KPYQFANSSFIIL	K	P	Y	-	Q	F	A	N	S	S	F	I	I
***Firmicutes bacterium* CAG:24**	NALTGRLGDFWNIVP	N	A	L	TG	R	L	G	D	F	W	N	I	V
***Firmicutes bacterium* CAG:65**	GSDRIDGAVSLLL	G	S	D	-	R	I	D	G	A	V	S	L	L

[1]strain 3_1_57FAA_CT1

Table S2. A lipocalin motif was found in a homologue of BrpA from *Clostridium sp.* KLE1755. Lipocalin proteins can bind hydrophobic molecules such as carotenoids and retinoids. The top ten BLASTP homologues to BrpA were aligned to compare these protein sequences and identify putative lipocalin motifs. The consensus motif is displayed on the top row of Table 4. Residues that match the consensus are shown in green and mismatches are shown in red
